# ﻿Description of a new species of *Chrysonotomyia* Ashmead from Houston, Texas, USA (Hymenoptera, Chalcidoidea, Eulophidae)

**DOI:** 10.3897/zookeys.1212.127537

**Published:** 2024-09-18

**Authors:** Brendan O’Loughlin, Pedro F. P. Brandão-Dias, Michael W. Gates, Scott P. Egan

**Affiliations:** 1 Department of BioSciences, Rice University, Houston, Texas, 77005, USA Rice University Houston United States of America; 2 Systematic Entomology Laboratory, USDA-ARS, c/o NMNH, Smithsonian Institution, 10th & Constitution Ave. NW, Washington, DC, USA Smithsonian Institution Washington DC United States of America

**Keywords:** Cynipidae, live oak, *Neuroterus* nr. *Bussae*, parasitoid, *Quercusvirginiana*

## Abstract

A new species of the genus *Chrysonotomyia* Ashmead, *Chrysonotomyiasusbelli***sp. nov.**, is described from the Rice University campus in Houston, Texas, USA. The species is a parasitoid emerging from Neuroterusnr.bussae galls in leaves of the southern live oak (*Quercusvirginiana*). This represents the 6^th^ species described from North America north of Mexico and the first in the world known to parasitize cynipid gall wasps. This discovery hints at an entire undiscovered niche between *Chrysonotomyia* parasitoids, cynipid gall wasps, and oaks in the Nearctic, which is a global biodiversity hotspot for oaks and cynipids. This new species description is complemented by mtDNA-COI-barcode data and information on the natural history of this species. We record host association, phenology, and report a leaf-scanning behavior performed by females, presumably to search for host galls. Modifications to the key of New World members of the genus (Hansson 2004) are included to integrate this new species.

## ﻿Introduction

*Chrysonotomyia* Girault, 1904 is a genus of parasitic wasps in the family Eulophidae (Hymenoptera) with close to 173 described species (Hansson 2004; Paniagua et al. 2009; Singh et al. 2022). The family Eulophidae is composed of parasitoid wasps, who are natural enemies of a wide range of insects (Marris and Edwards 1995; Triapitsyn 2005). Accordingly, members of the genus *Chrysonotomyia* are reported to attack a wide range of host insects (Table 1) but tend to specialize on a specific life stage, where they are found as parasitoids of the larvae of endophytophagous insects such as gall-inducers, leaf-miners, and stem-miners (Hansson 1990, 2004). The few known hosts of New World *Chrysonotoymia* are predominantly gall midges (Diptera, Cecidomyiidae), with single-species records from Torymidae (Hymenoptera) galls, Momphidae (Lepidoptera) leaf-miners, Psyllidae (Hemiptera) galls, and scale insects in the family Coccidae (Hemiptera) (Hansson 2004).

**Table 1.** Number of *Chrysonotomyia* species known to attack galls or endoparasitoids from each taxonomic family.

Order	Family	Number of associated *Chrysonotomyia* sp.
Diptera	Cecidomyiidae	27
Hemiptera	Coccidae	1
	Diaspidae	1
	Psyllidae	2
Hymenoptera	Cynipidae	1
	Torymidae	1
Lepidoptera	Momphidae	1
Galls of Unknown Taxonomic Affinity		10

While the genus has a near-cosmopolitan distribution, its center of diversity is in the Americas, specifically in the Neotropics (Hansson 2004). Hansson (2004) produced the latest revision of North and Central American *Chrysonotomyia*, with additional modifications to the key made by Paniagua et al. (2009). The vast majority of species cataloged by Hansson (2004) and Paniagua et al. (2009) were newly described from field-collected specimens, with a small number previously described by other authors. Notably, these species rarely have a known ecology and host record. Currently, according to the Universal Chalcidoidea Database UCD Community (2023), only five species of *Chrysonotomyia* have been recorded in North America north of Mexico – *C.aemilia* Girault, 1917; *C.auripunctata* Ashmead, 1894; *C.maculata* Delucchi, 1962; *C.phenacapsia* Yoshimoto, 1972; and *C.pherocera* Hansson, 2004.

Here, we describe the sixth species of *Chrysonotomyia* known from the United States, the first of the entire genus confirmed to attack cynipid gall wasps, and the first in a confirmed association with oaks in the genus *Quercus*. Specifically, we discovered *Chrysonotomyia* parasitoids emerging from leaf galls induced by Neuroterusnr.bussae Melika & Nicholls, 2021 (Hymenoptera, Cynipidae) on the southern live oak *Quercusvirginiana* Mill. (Fagaceae). The morphological species description is complemented by genetic barcode sequences and details of their natural history and phenology. We also include suggested modifications to the key by Hansson (2004) to incorporate the new species.

## ﻿Materials and methods

### ﻿Field collections and gall rearing

Individuals were reared from galls at the type locality, Houston, Texas, on the Rice University campus (29.717, −95.402) and collected as adults on the host plant, *Quercusvirginiana*. Currently, this is the only location from which the species has been confirmed. However, we believe it should be found within the range of its insect host on its host plant, found throughout the southern United States along the Gulf Coast (Cavender-Bares et al. 2015; Driscoe et al. 2019). For insect rearing from gall tissue, galls identified as Neuroterusnr.bussae on *Q.virginiana* were placed into holding containers in early March 2023, after which the new species shortly emerged later in May and early April. The term “Neuroterusnr.bussae” is used due to genetic data which suggests the species in Texas may be different from the one described from Florida (Egan Lab, unpublished data). Free-living adult individuals were also collected via aspirator from leaves of *Q.virginiana*. All individuals of the new species were preserved in 96% EtOH and frozen in a −80 °C ultrafreezer.

### ﻿Morphological descriptions and type material location

The description of the species was made under a Leica 205C stereomicroscope. Specimens were imaged using a Macropod using Canon EOS R6 equipped with a Canon 113 Zoom Lens EF 70–200mm and M Plan Apo 10× and 20× compound objective lenses. Images were then stacked in Zerene Stacker (v. 1.04 Build T2023–114 06–11–1120, Zerene Systems). Final images were processed in Adobe Photoshop 2023.

The holotype, syntype, and all paratypes were deposited in the National Museum of Natural History in Washington D.C (**USNM**).

### ﻿Complementing morphological taxonomy with molecular barcodes

Three individuals were selected for molecular barcoding: one male and one female that had emerged from galls (harvested 4/18/2022, emerged 5/6/2022 and 5/4/2022 respectively), and one female caught via aspirator directly from the host plant leaf (May 2023). Genetic material was extracted using DNeasy Blood and Tissue kits (Qiagen Inc., Valencia, CA). We used a pair of degenerate primers to amplify a segment of the mitochondrial cytochrome oxidase (mtDNA - COI) gene using standard PCR protocols (Smith et al. 2008). Primers used were COI pF2: 5′ – ACC WGT AAT RAT AGG DGG DTT TGG DAA – 3′ and COI 2437d: 5′ – GCT ART CAT CTA AAW AYT TTA ATW CCW G – 3′, developed by Simon et al. (1994) and modified by Kaartinen et al. (2010). Samples were sent to the University of Arizona for sequencing. We edited raw sequences and assembled forward and reverse reads using Geneious v. 6.1.8 (Kearse et al. 2012). The final sequences were 620, 622 and 631bp in length. We ran each sequence through the “identification request” module on the Barcode of Life Database (BOLD; Ratnasingham and Hebert 2007) to identify the highest percentage matches from previously identified taxa. All sequences were deposited in GenBank (accession numbers provided below).

### ﻿Abbreviations

Abbreviations follow the terminology used in Hansson (2004) and Paniagua et al. (2009).

**HE/MS/WM** Ratio of height of eye (HE), malar space (MS) and width of mouth opening (WM)

**POL/OOL/POO** Ratio of distance between posterior ocelli (POL), distance between the posterior ocellus and the compound eye (OOL) and the distance between occipital margin and posterior ocelli (POO)

**WH/WT** Ratio of width of head in dorsal view (WH), and the width of thorax (WT), measured across widest part

**LW/>LM/HW** Ratio of length of wing (LW), length of marginal vein (LM) and height of wing (HW)

**PM/ST** Ratio of length of postmarginal vein (PM) and the length of stigmal vein (ST)

**MM/LG** Ratio of length of mesosoma measured from anterior margin of pronotum to posterior margin of propodeum (MM) and the length of gaster (LG)

## ﻿Results

### ﻿Taxonomy

#### ﻿ Chrysonotomyia

Taxon classificationAnimaliaHymenopteraEulophidae

Ashmead, 1904

8A2D2336-7C05-5033-B9BD-704EF661CBD9

Chrysonotomyia Ashmead, 1904. Journal of the Linnean Society (Zoology) 25: 166.

##### Type species.

*Eulophusauripunctatus* Ashmead.

##### Diagnosis.

Subtorular sulci present; clypeus delimited laterally only (sometimes weakly so); occiput without vertical groove or weak fold between occipital margin and occipital foramen; postmarginal vein usually shorter (0.1–0.8×) than stigmal vein (but 2.3–3.2× as long in species group *neeigena*); midlobe of mesoscutum with one pair of setae (2–3 pairs in a few species); notauli poorly delimited or missing in posterior part; petiolar foramen rounded triangular; petiole very short, as a narrow band (as long as wide in a very few species); male phallobase: digitus with one spine (a few species with two spines (mainly the species in group *planiseta*), digitus is missing in species group *laeviscuta*) (Hansson 2004).

###### ﻿Species group *auripunctata*

**Diagnosis.** Midlobe of mesoscutum with one pair of setae; flagellomeres with short and asymmetric sensilla; digitus in male genitalia with one spine (Hansson 2004).

#### ﻿ Chrysonotomyia susbelli sp. nov.

Taxon classificationAnimaliaHymenopteraEulophidae

DD27BBF7-7157-5D21-BBA8-D815EEB64248

https://zoobank.org/9D4FE13C-E9C2-4A9E-B504-CB413DC9FD12

Figs 1 , 2

##### Diagnosis.

Mesosoma predominantly golden yellow with dark brown markings dorsally. Similar to *C.corynata* (Hansson, 2004) but differing in hue and dorsal patterning; dorsellum visible in dorsal view; antennae not distinctly clavate; flagellomere five dark brown; gaster with dark brown transverse bands, never more than two complete dorsally.

##### Description.

Length of body ♀ 1.0–1.2 mm, ♂ 0.9 mm.

**Figure 1.**
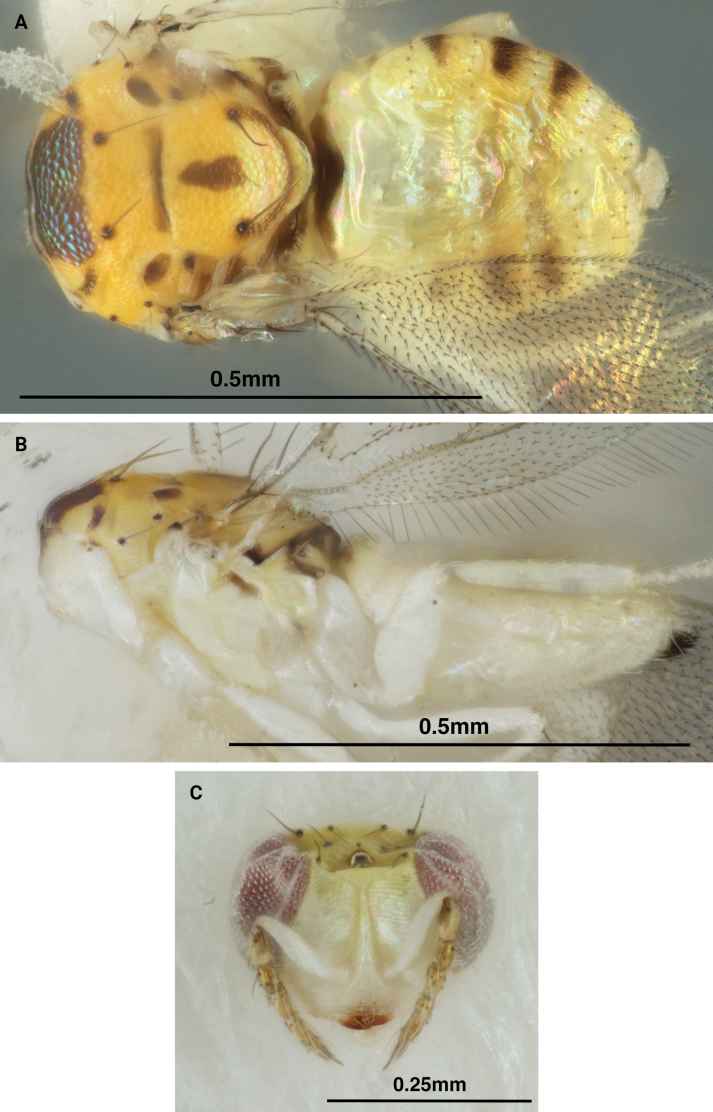
*Chrysonotomyiasusbelli* sp. nov. female holotype **A** dorsal habitus **B** lateral habitus **C** frontal view of head.

**Figure 2.**
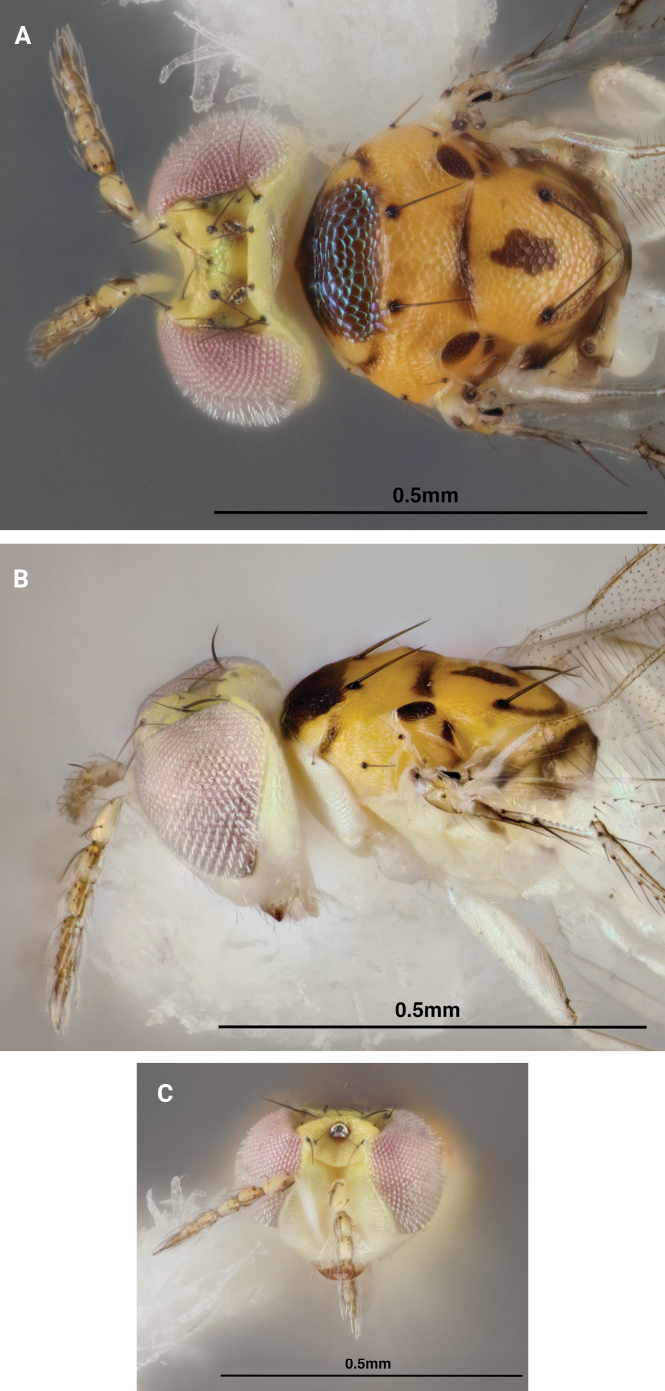
*Chrysonotomyiasusbelli* sp. nov. male syntype **A** dorsal habitus **B** lateral habitus **C** frontal view of head.

Mesosoma female: Mesoscutum golden yellow with the posterior midlobe occupied by a transverse, metallic brown band flanked by small transverse dark bands on the sidelobes, the metallic brown band may appear metallic green when viewed at certain angles. Axillae golden yellow with 2–3 areas of dark brown coloration. Scutellum golden yellow with median longitudinal dark band, anterior and posterior edges lined with dark transverse bands. Dorsellum golden yellow. Propodeum light yellow with a dark brown transverse band (Fig. 1A). Pronotum, prepectus, transepimeral sulcus, propodeal callus, lower mesepimeron and ventral mesosoma pale white, creating a distinct countershading between golden yellow dorsally and pale white ventrally. Transepimeral sulcus and lower mesepimeron often with thin, longitudinal bands of dark brown coloration (Fig. 1B). Mesosoma male: Same as female with dark bands significantly darker in color (Fig. 2A, B). Legs pale white in both sexes. Wings hyaline. Petiole dark brown.

Female gaster pale yellow with three incomplete dark transverse bands and apical ovipositor sheaths dark brown (Fig. 1A). Male gaster darker yellow, with two complete dark transverse bands preceded by an incomplete one (Fig. 2A).

Head light yellow apically, transitioning to pale white below. Eyes pink. Scape pale white, pedicel light yellow with apical two-thirds of inner surface dark brown, flagellomeres 1–4 yellow, flagellomere 5 brown (Figs 1C, 2C). Occiput light yellow, male sometimes with large dark macula.

Both male and female antennae with pale white verticillate setae (Figs 1C, 2C). Vertex with weak reticulation inside ocellar triangle, smooth outside triangle. Frontal suture weakly curved dorsad. Occipital margin carinate. Ratios of HE/MS/WM ♀ 2.5/1.5/1.0, ♂ 2.7/1.0/1.2; POL/OOL/POO ♀ 1.0/1.1/1.5, ♂ 4.9/2.7/1.0; WH/WT 1.1.

Mesoscutum and scutellum with weak and small meshed reticulation. Dorsellum small, convex, and smooth (Fig. 1A, 2A). Forewing with speculum closed below; without stigmal hair lines; radial cell bare. Ratios of LW/LM/HW 2.0/1.0/1.1; PM/ST 0.5.

Female gaster ovate. Ratio of MM/LG ♀ 1.0/1.1, ♂ 1.0/1.0

##### Biology.

**Figure 3.**
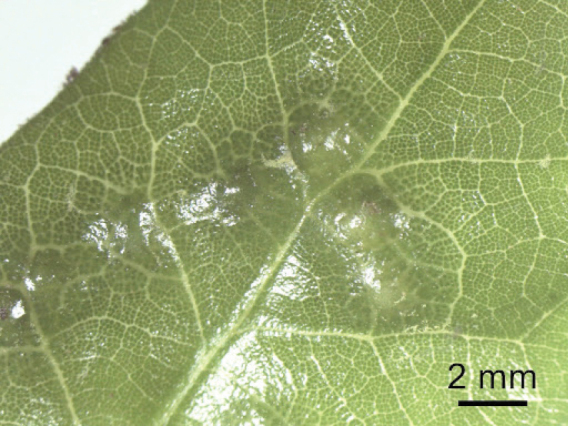
*Neuroterusnr.bussae* galls in leaves of the southern live oak (*Quercusvirginiana*) collected in Houston, Texas, U.S.A.

Known to parasitize Neuroterusnr.bussae (Fig. 3), a cynipid which forms galls on *Quercusvirginiana*. Preliminary genetic data and biogeographic patterns in this system suggest that the form of *Neuroterusbussae* present in Houston may not be the same species as the ones described from Florida, but this requires additional work. Thus, we conservatively refer to the insect host as Neuroterusnr.bussae (Egan lab, unpublished data). Live females were observed under both lab and field conditions. Adults always moved in a zigzag pattern, both in captivity and the wild. In the field, females were observed moving back and forth on upper leaf surfaces (Suppl. material 1). We hypothesize that this movement represents gall-searching behavior. Currently the species is only known from the Rice University campus in Houston, Texas, USA, but it likely follows the distribution of its host, Neuroterusnr.bussae on *Quercusvirginiana*.

##### Material examined.

***Holotype*** • United States: Houston, Harris County; 29.7179°N, 95.4048°W; 4.v.2022. ex. gall on *Quercusvirginiana*; coll. Pedro Brandão-Dias (1♀; USNMENT01928159). ***Syntype*** • same data as holotype; 10.v.2022 (1♂; USNMENT01928158). ***Paratypes*** • (1♀; USNM) same data as syntype; 25.iv.2022 • (1♀ 1♂; USNM) same data as syntype; 29.iv.2022 • (1♀; USNM) same data as syntype, 2.v.2022 • (5♀; USNM) same locality as holotype; 20.iv.2023–18.v.2023; on leaves of *Quercusvirginiana*, coll. Brendan O’Loughlin.

##### Molecular barcodes.

The three mtDNA-COI sequences were, on average, 99.4% identical to each other and, on average, 91.1% identical to *Chrysonotomyia* sp. PLACZ361-20 from Guanacaste, Costa Rica in the BOLD database. The sequences can be accessed through GenBank accession numbers PP468569, PP468570, and PP468571. See Suppl. material 2 for the exact mtDNA-COI sequences.

##### Phenology.

Adult *C.susbelli* sp. nov. were observed emerging from galls in the lab from 18 April to 10 May 2022, and we made additional observations of adult *C.susbelli* sp. nov. on the leaves of the southern live oak (*Q.virginiana*) from 20 April to 18 May 2023.

##### Etymology.

From Latin *sus belli*, roughly translating to “warpig”, in reference to the mascot of the Rice University dormitory Wiess College where the first author currently resides, whose official color is similar to the golden yellow of the dorsal mesosoma. Wiess College is named for Harry Carothers Wiess (1887–1948), one of the founders and one-time president of Humble Oil, whose generosity, with time, mind, and resources, greatly shaped the expansion of Rice University.

### ﻿Differential diagnostics

#### ﻿Morphologically similar species

**Figure 4.**
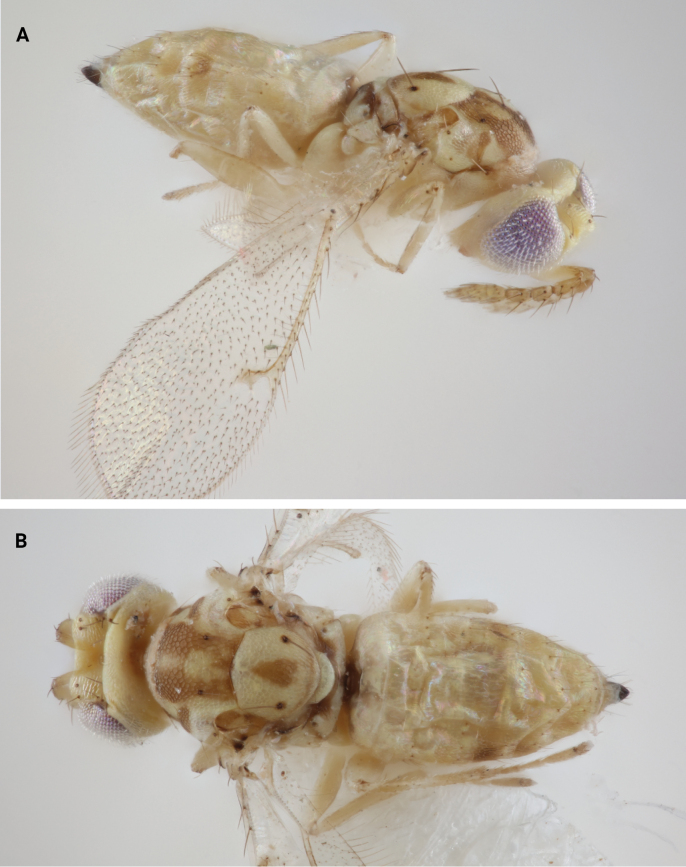
*Chrysonotomyiacorynata* Hansson, 2004 **A** lateral habitus **B** dorsal habitus.

*Chrysonotomyiasusbelli* sp. nov. most closely keys to *C.corynata* using the key by Hansson (2004). Based on Hansson’s (2004) morphological description of *C.corynata* and photos provided by Jim Woolley at the Texas A&M Entomological Collection, *C.corynata* can be distinguished from *C.susbelli* sp. nov. using the following features: dorsal mesosoma pale yellow rather than bright golden yellow (Fig. 4B); presence of a longitudinal rather than transverse band on the anterior mesoscutum (Fig. 4B); lower half of the body similar in color to upper half (Fig. 4A); female antennae distinctly clavate (Fig. 4A).

*Chrysonotomyiacorynata* is also geographically distant and only known from specimens collected in the Mexican states of Coahuila, Guanajuato, Michoacán, Oaxaca, Puebla and Zacatecas (Hansson 2004). Its ecology is unknown.

### ﻿Modification to the key by Hansson (2004)

To incorporate *C.susbelli* sp. nov. into the key in Hansson (2004), we suggest the following modifications:

**Table:** 

47(46)	Mesosoma completely yellowish-white; forewing with denser setation (as in fig. 284)	***C.crinipennis*** (♀)
–	Mesosoma with some parts brown; forewing with less dense setation (fig. 285)	**47a**
47a(47)	Flagellomeres similar in color to scape	***C.corynata***
–	Flagellomeres significantly darker than scape	***C.susbelli* sp. nov.**

#### ﻿Ecologically similar species

While all New World *Chrysonotomyia* with a reported host association parasitize galls (Hansson 2004), *C.susbelli* sp. nov. appears to be the first in the genus to attack galls induced by Cynipidae. One species formerly placed in the genus was recorded from Cynipid galls in Japan, but it was transferred to *Closterocerus* by Hansson (1999)—namely *Closteroceruscinctiventris* Ashmead, 1994 (Kamijo 1976). Additionally, the Universal Chalcidoidea database records *Chrysonotomyiaobesula* Boucek, 1986 as parasitizing cynipids (UCD Community 2023). However, Boucek’s original description records *C.obesula* only from Cecidomyiidae (Boucek 1986).

Of the 103 *Chrysonotoymia* previously described from North America, only 33 species (~32%) have known hosts (Hansson 2004; Paniagua et al. 2009). For reference, we provide a list of insect hosts (Table 1) and plant associates (Table 2) at the family level for the North American members of the genus, adapted from Hansson (2004), Paniagua et al. (2009), and data from *C.susbelli*. Note that several species are known from more than one gall and plant associate.

**Table 2.** Number of *Chrysonotomyia* species known to attack galls or endoparasites associated with each taxonomic plant family.

Plant Family	Number of associated *Chrysonotomyia* sp.
Aquifoliaceae	1
Cannabaceae	1
Cecropiaceae	5
Celastraceae	1
Chrysobalanaceae	2
Ericaceae	1
Fabaceae	6
Fagaceae	1
Lauraceae	1
Lecythidaceae	2
Loranthaceae	1
Malvaceae	1
Melastomataceae	2
Moraceae	4
Myrtaceae	2
Olacaceae	1
Piperaceae	2
Polygonaceae	2
Rhizophoraceae	1
Rosaceae	1
Rubiaceae	2
Sapindaceae	1
Smilaceae	1
Urticaceae	2
Verbenaceae	1
Vitaceae	1

#### ﻿Geographically proximate species

Five other *Chrysonotomyia* are known from the United States: *C.aemilia* Girault, 1917; *C.auripunctata* Ashmead, 1894; *C.maculata* Delucchi, 1962; *C.phenacapsia* Yoshimoto, 1972; and *C.pherocera* Hansson, 2004 (Hansson 2004, UCD Community 2023). Features useful for separating these from *C.susbelli* sp. nov. are given for each. Our modified key, above, works to distinguish *C.susbelli* sp. nov. from all other *Chrysonotomyia*, but we provide specific differences below.

*Chrysonotomyiaaemilia*. This species is known only from Florida (UCD Community 2023). It can be distinguished from *susbelli* sp. nov. by a long, lanceolate gaster and lack of dark coloration on the dorsal mesosoma (Hansson 2004). Known from unspecified Cecidomyiidae galls on *Eugeniafoetida* Pers. (Myrtaceae) (Burks 1979).

*Chrysonotomyiaauripunctata*. This species is known from Florida, Brazil, Costa Rica, Guatemala, Guyana, Mexico, Panama, St. Vincent, and Trinidad. It can be distinguished from *susbelli* sp. nov. by the scutellum hiding the dorsellum in dorsal view and the presence of two hair lines radiating from the stigmal vein (Hansson 2004). Within the United States it is only known to attack the cecidomyid *Ctenodactylomyiawatsoni* Felt, 1915 on *Coccoloba* sp. (Polygonaceae) (Burks 1979).

*Chrysonotomyiamaculata*. This species is known from Canada, Costa Rica, Honduras, and the USA (Florida, Maryland, New York, Ohio, Tennessee, and Texas). It can be distinguished from *C.susbelli* sp. nov. by its pale antennae, distinctly elongate female gaster, and partly to completely hairy radial cell (Hansson 1994, 2004). In the United States, it is known to attack *Pachypsyllaceltidismamma* and *Pachypsyllaceltidisvesicula* galls (Hemiptera, Aphalaridae) on *Celtis* sp. (Cannabaceae) (Burks 1979; Hansson 1994). There is a single record in Hansson (1994) of six specimens reared from a “Cynipid gall on Quercus leaf”. However, since it is a single occurrence and the insect host and host plant were not specified, this is uncertain until further evidence emerges.

*Chrysonotomyiaphenacapsia*. It can be distinguished from *susbelli* sp. nov. by the midlobe of the mesoscutum with two pairs of setae, and strong reticulation on the frons above the frontal suture (Hansson 2004). In the United States (California, Florida, and Texas), known to parasitize scale insects of the family Diaspidae (Hemiptera) on *Pinusjeffreyi* Balf., introduced *Pinuspinea* L. and *Picea* sp. (Pinaceae) (Yoshimoto 1972; Luck and Dahlsten 1974; Burden and Hart 1994).

*Chrysonotomyiapherocera*. Known from Costa Rica, Mexico, and the USA (Florida and Missouri). It is easily distinguishable from *C.susbelli* sp. nov. by the anterolateral edges of the vertex produced forward of the eyes, creating “horns” (Hansson 2004). Its ecology is unknown.

## ﻿Discussion

While *C.susbelli* sp. nov. has officially been recorded only from Houston, citizen-science observations on platforms such as Bugguide.net and iNaturalist hint at a possibly much broader distribution, and/or the presence of closely related undescribed species across the United States. There is one iNaturalist observation from Manitoba, Canada (https://www.inaturalist.org/observations/183137943) that appears to show a superficially similar *Chrysonotomyia* in association with the cynipid *Druonignotum* Bassett, 1881. Two other observations, one from Bugguide in Texas (https://bugguide.net/node/view/908461) and the other from iNaturalist in New York (https://www.inaturalist.org/observations/173105503), show a similar *Chrysonotomyia* associated with galls on *Vitis* spp. (Vitaceae). Whether these observations represent *C.susbelli* sp. nov. or a closely related species are difficult to tell without specimens and genetic data.

Our work suggests that there might be more undescribed *Chrysonotomyia* hiding among the 90+ oak species in the US and Canada and 180+ oak species in Mexico (POWO 2024), each with a diverse community of gall-forming cynipid wasps, which can range from 5–50 species per host (Cornell 1985). If just one *Chrysonotomyia* was associated with one leaf galling cynipid species, there could easily be other *Chrysonotomyia* still to be discovered across Mexico, the US, and Canada.

Interestingly, we also describe here a novel leaf-scanning behavior performed by females on the leaves of live oaks (Suppl material 1). In this behavior, females very quickly moved along the adaxial side of leaves in a zigzag pattern while tapping its antenna on the leaf surface. This behavior was observed in the field multiple times by the authors and performed always by females on the leaves of its host`s host, *Q.virginiana*. In the provided video, one can observe that the female stops at a leaf distortion superficially similar to its host gall and taps it repeatedly for better examination. This led us to hypothesize that this is a host-searching behavior performed by mated females looking to oviposit.

## Supplementary Material

XML Treatment for Chrysonotomyia

XML Treatment for Chrysonotomyia susbelli

## ﻿Additional information

### Author ORCIDs

Brendan O'Loughlin https://orcid.org/0009-0002-9925-8492

Pedro F. P. Brandão-Dias https://orcid.org/0000-0001-8583-0617

Michael W. Gates https://orcid.org/0000-0002-5760-1371

### Data availability

All of the data that support the findings of this study are available in the main text or Supplementary Information.

## Supplementary materials

Supplementary material 1 Leaf scanning behavior of *Chrysonotomyiasusbelli* sp. nov. Data type mp4 https://binary.pensoft.net/file/1135989This dataset is made available under the Open Database License (http://opendatacommons.org/licenses/odbl/1.0/). The Open Database License (ODbL) is a license agreement intended to allow users to freely share, modify, and use this Dataset while maintaining this same freedom for others, provided that the original source and author(s) are credited.Brendan O’Loughlin, Pedro F. P. Brandão-Dias, Michael W. Gates, Scott P. Egan

Supplementary material 2 Raw COI sequences of *Chrysonotomyiasusbelli* sp. nov. Data type fasta https://binary.pensoft.net/file/1135990This dataset is made available under the Open Database License (http://opendatacommons.org/licenses/odbl/1.0/). The Open Database License (ODbL) is a license agreement intended to allow users to freely share, modify, and use this Dataset while maintaining this same freedom for others, provided that the original source and author(s) are credited.Brendan O’Loughlin, Pedro F. P. Brandão-Dias, Michael W. Gates, Scott P. Egan
